# The genetics of indirect ecological effects—plant parasites and aphid herbivores

**DOI:** 10.3389/fgene.2014.00072

**Published:** 2014-04-08

**Authors:** Jennifer K. Rowntree, Sharon E. Zytynska, Laurent Frantz, Ben Hurst, Andrew Johnson, Richard F. Preziosi

**Affiliations:** Environment and Ecology Research Group, Faculty of Life Sciences, University of ManchesterManchester, UK

**Keywords:** indirect ecological effects, community genetics, *Rhinanthus minor*, *Sitobion avenae*, eco-evolutionary feedbacks

## Abstract

When parasitic plants and aphid herbivores share a host, both direct and indirect ecological effects (IEEs) can influence evolutionary processes. We used a hemiparasitic plant (*Rhinanthus minor*), a grass host (*Hordeum vulgare*) and a cereal aphid (*Sitobion avenae*) to investigate the genetics of IEEs between the aphid and the parasitic plant, and looked to see how these might affect or be influenced by the genetic diversity of the host plants. Survival of *R. minor* depended on the parasite's population of origin, the genotypes of the aphids sharing the host and the genetic diversity in the host plant community. Hence the indirect effects of the aphids on the parasitic plants depended on the genetic environment of the system. Here, we show that genetic variation can be important in determining the outcome of IEEs. Therefore, IEEs have the potential to influence evolutionary processes and the continuity of species interactions over time.

## Introduction

Ecological communities comprise complex networks of interacting species that influence each other via many direct and indirect effects. In an ecological context, direct effects are those that one species exerts on another species with which it directly interacts: e.g., plant-herbivore interactions (Agrawal et al., 2006). Indirect ecological effects (IEEs) are mediated via a third species (Wootton, 1994): e.g., host plant effects on a predator mediated via the herbivorous prey (Astles et al., 2005). Both direct and indirect effects are thought to play important roles in evolutionary processes via the promotion or constraint of phenotypic evolution (Wootton, 1994), although specific evidence for this is limited (Strauss and Irwin, 2004).

Genetic diversity is the key ingredient linking ecological and evolutionary processes, and sufficient genetic diversity is required if ecological interactions (including IEEs) are to affect the evolution of phenotypes. The discipline of community genetics seeks to provide a framework that allows us to investigate the influence of intraspecific genetic variation in one, or more, species on the phenotype of other interacting species (Antonovics, 1992; Rowntree et al., 2011b). Work has shown that there is a genetic basis for many direct effects (e.g., Whitham et al., 2003; Johnson and Agrawal, 2005; Zytynska et al., 2011) and IEEs (e.g., Bailey et al., 2006; Johnson, 2008). We also know that variation in the response of a focal species to an indirect effect can have a genetic component (Astles et al., 2005), and that incorporation of genetic variation in more than one species in multi-trophic systems can change the direction and magnitude of an indirect effect, in terms of the fitness of the focal species (Tétard-Jones et al., 2007). Therefore, there is real potential for IEEs to influence evolutionary processes, but, the real impact of genetic variation in multiple components of a trophic system, incorporating direct and indirect effects, remains under-explored.

Infection by a parasitic plant can directly influence the success of a host plant by dramatically changing host biomass accumulation (Cameron et al., 2005). Genetically-based variation within host, parasitic plant or both, has the potential to change the outcome of the direct interaction in terms of host biomass or parasite size, biomass, and fecundity (Mutikainen et al., 2000; Ahonen et al., 2006; Rowntree et al., 2011a). Furthermore, genetic diversity in the host population can influence community level response to infection, with genetically impoverished host communities potentially being less resistant to the parasite (Spielman et al., 2004). Host and parasitic plants can also indirectly influence herbivores feeding on the other plant by either increasing (e.g., Ewald et al., 2011) or decreasing (e.g., Marvier, 1996) fitness. Where parasitic plants and herbivores share a host, the indirect effects of herbivores on the parasites have not often been recorded, and we do not know if such indirect effects have the potential to shape evolutionary processes.

We used the hemi-parasitic plant *Rhinanthus minor*, a grass host (*Hordeum vulgare*) and a cereal aphid (*Sitobion avenae*) to investigate the genetics of IEEs (subsequently GIEEs) from the aphids to the hemi-parasite. Specifically, we wanted to see if there was an interaction between aphid genotype and *R. minor* population that influenced the survival and reproductive success of the parasitic plant. Although *R. minor* is not a problem in agricultural systems due to modern management practices, it does commonly infect grasses and the *Rhinanthus-Hordeum* experimental system is well-established (Seel and Jeschke, 1999; Jiang et al., 2004). We also looked at how levels of genetic diversity within the host community might influence the interactions between hosts, parasites and herbivores. We incorporated genetically-based intra-specific variation in both the aphids and the parasitic plant, and used either genetically uniform or genetically diverse hosts. We asked: (1) if there are indirect effects of the aphids on the parasitic plants; (2) if there is a genetic basis to any indirect effects observed (i.e., if different genotypes of aphid differentially impact the parasitic plants); (3) if there is a genetic basis for the response of an organism to an indirect effect (i.e., if parasite populations vary in response to the aphids); (4) if the presence of genetic diversity within the host plant mediates any indirect effects observed. Our experiment was designed to investigate whether genetic variation in any of the component species of a tri-trophic system was important in defining the indirect interactions between aphid herbivores and parasitic plants competing indirectly for nutrients via a common host. It also enabled us to determine the relative importance of genetic variation within a species and genetic interactions among species on the survival, reproductive potential and biomass production of the plants.

## Materials and methods

We obtained seeds of six doubled haploid barley (*Hordeum vulgare* L.) genotypes (Morex, Steptoe, Blenheim, Kym, Oregon Wolfe Dominant, Oregon Wolfe Recessive) from P. Hayes (Oregon State University, USA), which were bulked prior to use. We obtained seeds from two populations of *Rhinanthus minor* L.: the first originating in Somerset from Emorsgate Seeds (Kings Lynn, Norfolk, UK) and the second originating in Moray near Inverness from Scotia seeds (Brechin, Angus, UK). We obtained four cereal aphid (*Sitobion avenae*) genotypes (DAV95, CLO7, HF92a, H1) from Rothamstead Research, Harpenden, UK. Aphid genotypes were maintained and bulked on “pearl” barley to avoid conditioning effects. Two of the aphid genotypes were brown (HF92a, CLO7) and two were green (H1, DAV95) and we always paired one brown genotype with one green genotype to facilitate identification. This gave us a total of four aphid treatments (H1-HF92a; H1-CLO7; DAV95-HF92a; DAV95-CLO7).

We germinated barley seeds in the dark at 23°C and then transplanted them into pots (15 cm dia.) filled with horticultural sand (Keith Singleton Horticulture, Cumbria, UK). Barley seedlings were planted six to a pot and positioned in a circle around the edge. We used two diversity treatments: a genetically uniform treatment, where all six barley plants in a pot were the same genotype; and a genetically diverse treatment, where each barley plant was a different genotype. Genetically uniform pots were planted for each genotype and subsequent analyses combined all single genotype pots for the genetically uniform treatment. Treatments were randomly assigned to pots, as was position and genotype of the barley plants in the genetically diverse treatment.

We surface-sterilized *R. minor* seeds (1% v/v sodium hypochlorite solution, 3 min) and germinated them in the dark at 4°C (see Rowntree et al., 2011a for details). *Rhinanthus minor* is a generalist parasite with the propensity to attach to multiple hosts simultaneously (Gibson and Watkinson, 1989). Our aim was to start with three parasites successfully attached to one or more host plants. However, as not all seedlings are successful in attaching to a host we planted six *R. minor* seedlings (1–2 cm radicles) per pot. Parasite seedlings were planted in a circle in the center and positioned approximately 2 cm away from the barley plants. We used two populations of *R. minor* and a no *R. minor* control for each barley diversity and aphid-pair treatment. Plants were grown in a greenhouse (15–25°C) with supplementary lighting (16:8 photoperiod) and watered every day with ¼ strength Hoagland's solution (Hoagland and Arnon, 1950). The amount of nutrient solution added to the pots differed over time depending on the requirements of the plants, but was always consistent among pots on a single day. If the pots remained dry (i.e., if outside conditions were sunny and warm), we supplemented with water. In total, we planted 1230 germinated *R. minor* seeds in 205 pots and 1872 barley seedlings in 312 pots.

Two weeks after planting we counted all emerged seedlings of *R. minor.* Five weeks after planting we scored *R. minor* plants for morphological characteristics indicating host attachment and counted the number of attached plants (see Klaren and Janssen, 1978). At this point we reduced the number of *R. minor* to three per pot. We removed unattached plants preferentially and then chose which of the remaining individuals to remove randomly. Any pots without three attached *R. minor* were removed from the study leaving a total of 277 pots, 169 with *R. minor*. This gave us 8–12 pots per *Rhinanthus*-aphid combination in the genetically diverse treatment and 8–15 pots per *Rhinanthus*-aphid combination in the genetically uniform treatments.

Aphids were added 6 weeks after planting (±1 day). We put six fourth-instar or adult individuals of two aphid genotypes (one brown, one green) in a 6 cm petri dish (12 aphids per pot) and placed it open in the center of the pots. We used two aphid genotypes per replicate pot as one of the goals of the experiment was to investigate intraspecific competition among the aphids. These results have been previously reported (Zytynska et al., 2014) and are not included in our analysis here. We covered the plants and pots with fine mesh bags (Insectopia, Austrey, Warwickshire, UK) to ensure that aphids could not escape, but remained free to move among all plants within a treatment. Two weeks later (±1 day), after sufficient time to reproduce, we counted the numbers of each genotype of aphid on every plant in each pot. Finally, we removed the covers and all aphids from the plants by hand.

Thirteen weeks after planting, we counted the number of flowering *R. minor* individuals per pot to assess survival of potentially reproducing plants. We also counted the number of buds, flowers and seedpods (reproductive structures) per flowing plant and combined these as a measure of reproductive fitness. By the end of the experiment there were 95 pots in the *R. minor* treatment level containing a total of 206 flowering parasitic plants. Finally, we harvested each barley plant separately, dried them at 60°C for at least 2 days and measured shoot dry weight.

### Data analysis

**Figure 1:**
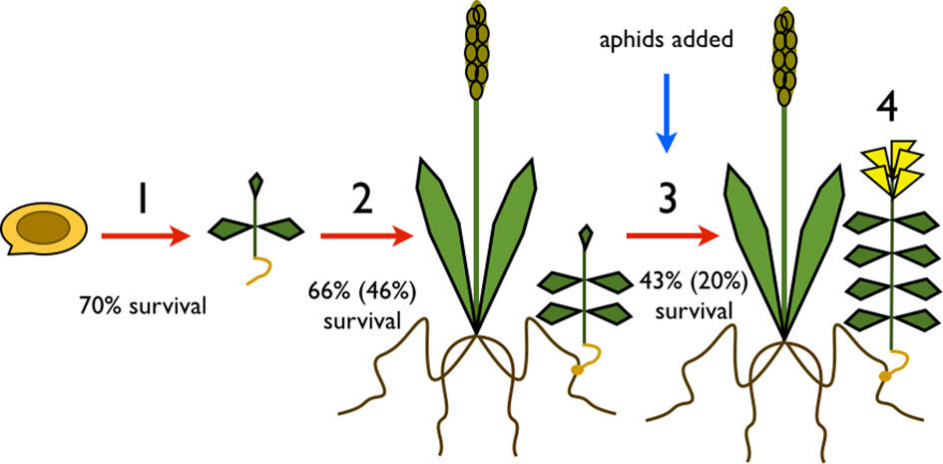
**Key life stages of *Rhinanthus minor* measured: (1) between germination and seedling emergence; (2) between emergence and attachment to a host; (3) post attachment through to production of flowers; (4) numbers of buds, flowers, and seedpods (reproductive structures) produced.** Conditional survival rates during each life stage are shown and are dependent on successful survival of the previous life stage. Absolute survival rates are shown in brackets.

We analyzed survival and fitness of individual *R. minor* plants at a series of key life history stages: (1) between germination and seedling emergence (emergence data); (2) between emergence and attachment to a host (attachment data); (3) attachment through to production of flowers (flowering data); and (4) the total number of buds, flowers and seedpods produced (Figure 1). The number of plants at each life history stage depended on successful survival of the previous stage. Therefore, the number of plants naturally declines for subsequent analyses. We used generalized linear mixed models with a binary distribution, a logit link function and pot as a random factor to analyse the survival data. *Rhinanthus minor* population (Somerset or Inverness) and barley diversity (uniform or diverse) were fixed factors in the analyses of emergence and attachment data, and interactions were included in the model. Fixed factors in the analysis of flowering data were *R. minor* population, barley diversity, green aphid genotype (H1 or DAV95) and brown aphid genotype (HF92a or CLO7). The final total number of aphids for each pot was used as a covariate and all possible interactions were included in the model. We corrected for variation in the time for aphid reproduction (either 14 or 15 days) by dividing final total number of aphids by number of days for each pot. The total number of buds, flowers and seedpods produced by each *R. minor* plant that survived were analyzed using a single generalized linear mixed model with a negative binomial distribution, a log link function and pot as a random factor. *Rhinanthus minor* population, barley diversity, green aphid genotype and brown aphid genotype were included as fixed factors, total number of aphids (corrected as above) was used as a covariate and all possible interactions were included in the model.

Prior to analysis, individual barley shoot dry weight data were square-root transformed. Q-Q plots of raw data, natural log transformed data and square root transformed data showed the latter to confirm best to normality. The shoot dry weight from individual barley plants was analyzed with a linear mixed model where pot was included as a random factor. *Rhinanthus minor* presence, barley diversity, green aphid genotype and brown aphid genotype were included as fixed factors, total number of aphids (corrected as above) and the final number of *R. minor* plants per pot were included as covariates. All possible interactions were included in the model. In order to test the effect of barley genotype on shoot dry weight, we subdivided the data by diversity treatment and reanalyzed using similar statistical models but replacing barley diversity with barley genotype. Data were split and reanalyzed as the experimental design did not allow us to incorporate genotype into the full model.

All analyses used the R statistical platform version 3.0.2 (R Core Team, 2013). Analyses of the survival and total bud, flower and seedpod data were performed using the glmmadmb function in the glmmADMB package (Fournier et al., 2012; Skaug et al., 2013). Analyses of the barley shoot dry weight was performed using the lmer function in the lme4 package (Bates et al., 2014). Significance values were calculated from Wald chi square tests using the Anova function in the car package (Fox and Weisberg, 2011). As part of the goal of the study was to uncover any potential interactions among treatments, we used a full model approach rather than a model simplification strategy (Crawley, 2012).

## Results

Only 20% of germinated seeds survived to produce flowers. When we broke survival down by the different life stages (Figure 1), 70% emerged above ground to produce seedlings. Emergence was higher in the Inverness population at 76% compared to the Somerset population at 64%. There was a significant effect of *R. minor* population on emergence (*X*^2^ = 4.87, *p* = 0.03), but no effect of host diversity (*X*^2^ = 0.008, *p* = 0.93) and no effect of the interaction between *R. minor* population and host diversity (*X*^2^ = 0.55, *p* = 0.46; see Table 1 for survival rates and Tables S1a and S1b for more test details). Of the plants that emerged, 66% successfully attached to a host plant but there were no significant effects of either *R. minor* population (*X*^2^ = 0.01, *p* = 0.91) or host diversity (*X*^2^ = 3.23, *p* = 0.07) and no significant interaction between the two (*X*^2^ = 2.32, *p* = 0.13; see Table 2 for survival rates and Tables S2a and S2b for more test details).

**Table 1:** **Percentage of germinated *R. minor* seed that emerged as seedlings by treatment groups**.

**Diversity**	***R. minor* population**	**Number Alive:Dead**	**% Germination**
Uniform	Inverness	256:80	**76**
	Somerset	196:122	63
Diverse	Inverness	224:70	**76**
	Somerset	188:94	67

Values in bold are greater than the overall survival rate of 70%.

**Table 2:** **Percentage of *R. minor* seedlings that successfully attached to a host plant following emergence (conditional attachment) and germination (absolute attachment) by treatment groups**.

**Diversity**	***R. minor* population**	**Number Alive:Dead**	**% Attachment (conditional)**	**% Attachment (absolute)**
Uniform	Inverness	187:69	**73**	**55**
	Somerset	124:72	63	40
Diverse	Inverness	145:79	65	**49**
	Somerset	122:66	65	44

Values in bold are greater than the overall conditional attachment rate of 66% and the overall absolute attachment rate of 46%.

Aphids were added between attachment and flowering of the *R. minor* (Figure 1) and although aphids were free to move around all plants within a pot, we only found aphids on the barley and did not find any on the *R. minor* plants themselves. Thus, any effects of the aphids on the *R. minor* were occurring indirectly via the host plants.

Subsequent *R. minor* survival until flowering was 43% overall but varied considerably across the treatment groups (Table 3). There was a significant four-way interaction between host diversity, *R. minor* population, green aphid genotypes and brown aphid genotypes (*X*^2^ = 4.20, *p* = 0.04; see Tables S3a and S3b for more test details), but no other significant effects. There were no significant effects of any of the factors tested on the number of *R. minor* seedpods (see Table 4 for median values and Tables S4a and S4b for more test details).

**Table 3:** **Percentage of *R. minor* plants that survived until flowering following successful attachment (conditional survival) and germination (absolute survival) by treatment groups**.

**Diversity**	***R. minor population***	**Green aphid genotype**	**Brown aphid genotype**	**Number Alive: Dead**	**% Survival (conditional)**	**% Survival (absolute)**
Uniform	Inverness	Dav95	CLO7	15:17	**47**	**26**
			HF92a	12:24	33	18
		H1	CLO7	21:24	**47**	**26**
			HF92a	14:22	39	**21**
	Somerset	Dav95	CLO7	21:11	**66**	**26**
			HF92a	7:16	30	12
		H1	CLO7	14:10	**58**	**23**
			HF92a	19:11	**63**	**25**
Diverse	Inverness	Dav95	CLO7	14:18	**44**	**22**
			HF92a	8:21	28	14
		H1	CLO7	8:25	24	12
			HF92a	15:21	42	**21**
	Somerset	Dav95	CLO7	11:22	33	15
			HF92a	19:11	36	16
		H1	CLO7	8:14	36	16
			HF92a	10:17	37	16

Values in bold are greater than the overall conditional survival rate of 43% and the overall absolute survival rate of 20%.

**Table 4:** **Median numbers of buds, flowers, and seedpods produced by the *R. minor* plants that survived until flowering by treatment groups**.

**Diversity**	***R. minor* population**	**Green aphid genotype**	**Brown aphid genotype**	**Median**	**25/75th Quartiles**
Uniform	Inverness	Dav95	CLO7	11.0	7.0/23.0
			HF92a	8.0	4.5/14.5
		H1	CLO7	**13.0**	7.0/20.0
			HF92a	8.0	4.25/22.5
	Somerset	Dav95	CLO7	**15.0**	12.0/19.0
			HF92a	**16.0**	8.0/21.5
		H1	CLO7	**17.5**	12.25/21.75
			HF92a	8.0	6.5/14.0
Diverse	Inverness	Dav95	CLO7	8.5	0.5/16.25
			HF92a	4.5	0.75/13.0
		H1	CLO7	**14.0**	10.0/16.75
			HF92a	12.0	6.5/22.0
	Somerset	Dav95	CLO7	12.0	4.0/19.0
			HF92a	12.0	7.5/21.0
		H1	CLO7	10.5	8.25/13.5
			HF92a	3.5	0.25/15.75

Numbers in bold are greater than the overall median value of 12.0 (6/40).

Barley shoot dry weight was significantly reduced by the presence of *R. minor* (*X*^2^ = 17.25, *p* < 0.0001) and there was a significant negative relationship between dry weight and the number of *R. minor* plants (*X*^2^ = 42.18, *p* < 0.0001). There was a significant positive relationship between dry weight and the total number of aphids in the pot (*X*^2^ = 184.60, < 0.0001) and also a significant effect of brown aphid genotype where genotype HF92a had a greater positive impact on dry weight than genotype CLO7 (*X*^2^ = 4.15, *p* = 0.04; see Tables S5a and S5b for more test details).

Analysis of barley genotype effects was only possible by dividing the data by host plant diversity treatment. In the uniform diversity treatment, all of the barley plants in a single pot were of the same genotype and therefore in these pots it was not possible for other barley genotypes to influence barley shoot biomass. In these pots, barley genotype (*X*^2^ = 74.07, *p* < 0.0001), brown aphid genotype (*X*^2^ = 5.79, *p* = 0.02), *R. minor* presence (*X*^2^ = 5.23, *p* = 0.02), the number of *R. minor* per pot (*X*^2^ = 33.43, < 0.0001) and total aphid number (*X*^2^ = 155.53, *p* < 0.0001) were significant factors affecting barley shoot dry weight and there was a significant interaction between barley genotype, green aphid genotype and brown aphid genotype (*X*^2^ = 12.49, *p* = 0.02).

In the diverse barley treatment, each barley plant in a pot was a different genotype. Therefore, barley shoot biomass could be influenced by competition among barley genotypes. In these pots, there was a significant effect of barley genotype (*X*^2^ = 504.51, *p* < 0.0001), *R. minor* presence (*X*^2^ = 21.54, *p* < 0.0001), the number of *R. minor* per pot (*X*^2^ = 17.73, *p* < 0.0001) and the total number of aphids (*X*^2^ = 90.65, *p* < 0.0001), but no effect of brown aphid genotype (*X*^2^ = 0.2774, *p* = 0.60) on barley shoot dry weight. There was, however, a significant interaction between barley genotype and brown aphid genotype (*X*^2^ = 22.77, *p* = 0.0004; see Tables S6 and S7 for more details).

## Discussion

We collected data on the survival and reproductive fitness of the annual parasitic plant *R. minor* over four key life stages in the context of genetically variable multi-trophic species interactions. We found that the highest percentage of plants died post attachment and after addition of aphids to the host. Survival during this life stage was dependent on the genetic context of all species involved. Previously, we have demonstrated that aphid distribution across host plants is influenced by the presence of *R. minor*, and when present, by genetic diversity within the *R. minor* infecting the hosts (Zytynska et al., 2014). In this study we show that aphid genotype can have a reciprocal effect on the survival of the parasitic plant. Thus, our experimental system demonstrates that genetically-based feedbacks can occur (sensu Genung et al., 2011) between indirect competitors. Specifically, we show that the population of *R. minor* influences the distribution of aphid genotypes across the host plants, and that aphid genotypes, in combination with host genetic diversity, differentially influence the survival of individuals from two populations of *R. minor*.

We know from previous studies that genotype by genotype interactions can influence the direct interactions among species with important consequences for the outcome of, among other things, plant-herbivore (Tétard-Jones et al., 2007), host-parasite (Lambrechts et al., 2005; Salvaudon et al., 2005; Rowntree et al., 2011a), and plant-plant (Fridley et al., 2007) interactions. They also have the potential to influence the evolutionary and coevolutionary trajectories of the species involved (Thompson, 2013). Genetic variation within a focal species can also indirectly affect other species in the wider associated community (Fritz, 1995; Irwin, 2006; Johnson, 2008; Schädler et al., 2010) and determine how a particular species responds to an IEE (Astles et al., 2005). Previous studies have demonstrated indirect effects of herbivores on *R. minor* (Bass et al., 2010). However, to our knowledge, this is the first time that GIEEs have been shown to be an important factor determining the fitness of a parasitic plant. Moreover, in combination with our previous analysis we demonstrate that there are reciprocal GIEEs between the aphids and the parasitic plant (Zytynska et al., 2014). This means that GIEEs can play potentially important roles as selective agents in communities of interacting species (Ter Horst, 2010), and that there is potential for coevolution amongst indirectly interacting organisms.

We found similar levels of mortality for *R. minor* during the two initial life stages we examined (germination-emergence; emergence-host attachment). During these early life stages, only population of origin influenced *R. minor* survival, and then only during the first life stage. Population level differences do not necessarily indicate a genetic basis of an effect, because maternal and other non-additive effects cannot be excluded. However, when plants are grown in a common environment, population differences are a strong indicator of a genetic influence on the traits under investigation (Krebs, 2001), in this case, early survival of *R. minor*.

Aphids were added to the system following attachment of the parasitic plant to a host and prior to flowering. Mortality was far greater at this stage (attachment-flowering) compared to the two previous life history stages, suggesting that selection on *R. minor* was stronger post attachment to a host. This may indicate strong indirect competition with the aphid herbivores sharing the host plants, but we were unable to test for this directly, as we did not have treatments without aphids. Alternatively, mortality rates could have increased at this life stage due to the addition of the aphid mesh-cages to all of the pots. Previous work has demonstrated that *R. minor* is intolerant to shading and low light levels (Tesitel et al., 2011), which the aphid cages will have influenced. However, although shading was a confounding factor, it was consistent across treatments and does not explain the differential influence of the aphid genotypes on survival of the parasitic plant. Nor does it explain the decreased survival of *R. minor* with increasing host genetic diversity. Rather, this indicates that higher levels of genetic diversity in the host plants likely confer host community resistance to infection by the parasitic plant (cf. Spielman et al., 2004; Kaunisto and Suhonen, 2013).

The high levels of mortality following attachment of the parasite to a host plant and prior to flowering were unexpected, as successful host attachment should facilitate survival of the parasitic plant. The most common measures of fitness of *R. minor* in previous studies have been size, biomass or flower and seed production (Cameron et al., 2006; Rowntree et al., 2011a; Tesitel et al., 2011), and mortality rates of individuals are rarely reported. We found no effect of any of the factors tested on the combined production of buds, flowers and seedpods and, in fact, the majority of plants died before they were able to set seed. Although seed set must be the ultimate measure of reproductive success for an individual, we suggest that as single measures, the above traits are rather poor indicators of fitness in *R. minor* and that survival is also crucial to monitor. If, as is indicated here, there is differential survival of plant genotypes prior to flowering and seed set, then there is potential for GIEEs to result in diverging levels of fitness, despite the lack of difference observed in seedpod production.

Host plant shoot biomass was most strongly influenced by the level of infection by both the parasitic plant and the aphid herbivores, although we also detected an effect of the brown aphid genotype. Previously, we have found genetically based variation within both *R. minor* and the aphid *S. avenae* to influence host plant traits (Tétard-Jones et al., 2007; Rowntree et al., 2011a), although not in combination with each other. Much of our earlier work has demonstrated that the outcome of complex species interactions cannot be predicted by more simplistic interactions among pairs of components (Tétard-Jones et al., 2007; Zytynska et al., 2010) and we reach a similar conclusion here. We also found no evidence that the GIEEs between the aphids and the *R. minor* had any impact on the host plants themselves. Rather, genotype by genotype interactions between the barley and the aphid herbivores had a greater impact on barley shoot biomass.

When biomass data from the diverse barley treatments were analyzed separately, we found a strong effect of barley genotype as well as the level of infection by the aphids and *R. minor*. There were also higher order interactions between aphid and barley genotypes. This suggests that in a diverse genetic population of hosts, interactions between individual genotypes and interacting species may depend on the genotypes of neighboring hosts. Mutic and Wolf (2007) demonstrated the importance of neighbor genotype on multiple plant traits, including growth, and here we show that interactions among neighbor genotypes of plants have the potential to change the genetic interactions between a host plant and its aphid herbivores.

Our experiments include multiple genotypes and populations of multiple species, resulting in complex statistical models with many terms. As our intention was to test for, and identify, significant interactions we have not used a model simplification approach in our analyses (Crawley, 2012). In addition, we have used mixed models for hypothesis testing. This approach comes with the caveat that individual higher order interactions must be interpreted with caution. However, it is notable that our main finding that *R. minor* fitness is influenced by a higher order interaction between *R. minor* population, aphids and host genotype mirrors the results of Zytynska et al. (2014) where aphids were also influenced by these same higher order interactions.

In conclusion, we show that GIEEs of aphid herbivores on parasitic plants have the potential to influence community structure by affecting plant survival rates. Our work further demonstrates, and supports the findings of previous studies showing the importance of the genetic background of an ecological community on the performance of component species (Fridley et al., 2007; Whitlock et al., 2007, 2010). The value of within-species genetic variation on community and ecosystem processes is still largely unknown in systems without a single dominant long-lived plant (e.g., Whitham et al., 2006; Barbour et al., 2009). We show that variation within shorter-lived plants can influence the current community, and that effects on survival and reproduction could indeed impact the future generations and thus evolution in ecological communities.

### Conflict of interest statement

The authors declare that the research was conducted in the absence of any commercial or financial relationships that could be construed as a potential conflict of interest.

## References

[B1] Agrawal A. A. Lau J. A. Hamback P. A. (2006). Community heterogeneity and the evolution of interactions between plants and insect herbivores. Q. Rev. Biol. 81, 349–376 10.1086/511529 17240728

[B2] Ahonen R. Puustinen S. Mutikainen P. (2006). Host use of a hemiparasitic plant: no trade-offs in performance on different hosts. J. Evol. Biol. 19, 513–521 10.1111/j.1420-9101.2005.01024.x 16599927

[B3] Antonovics J. (1992). Toward community genetics, in Plant Resistance to Herbivores and Pathogens - Ecology, Evolution and Genetics, eds Fritz R. S. Simms E. L. (Chicago, IL: The University of Chicago Press), 426–449

[B4] Astles P. A. Moore A. J. Preziosi R. F. (2005). Genetic variation in response to an indirect ecological effect. Proc. Biol. Sci. 272, 2577–2581 10.1098/rspb.2005.3174 16321778 PMC1559979

[B5] Bailey J. K. Wooley S. C. Lindroth R. L. Whitham T. G. (2006). Importance of species interactions to community heritability: a genetic basis to trophic-level interactions. Ecol. Lett. 9, 78–85 10.1111/j.1461-0248.2005.00844.x 16958871

[B6] Barbour R. C. Forster L. G. Baker S. C. Steane D. A. Potts B. M. (2009). Biodiversity consequences of genetic variation in bark characteristics within a foundation tree species. Conserv. Biol. 23, 1146–1155 10.1111/j.1523-1739.2009.01247.x 19459892

[B7] Bass K. A. John E. A. Ewald N. C. Hartley S. E. (2010). Insect herbivore mortality is increased by competition with a hemiparasitic plant. Funct. Ecol. 24, 1228–1233 10.1111/j.1365-2435.2010.01743.x

[B8] Bates D. Maechler M. Bolker B. Walker S. (2014). lme4: Linear Mixed-Effects Models Using Eigen and S4. R package version 1.0–6. Available online at: http://CRAN.R-project.org/package=lme4

[B9] Cameron D. D. Coats A. M. Seel W. E. (2006). Differential resistance among host and non-host species underlies the variable success of the hemi-parasitic plant *Rhinanthus minor*. Ann. Bot. 98, 1289–1299 10.1093/aob/mcl218 17008350 PMC2803582

[B10] Cameron D. D. Hwangbo J. K. Keith A. M. Geniez J. M. Kraushaar D. Rowntree J. (2005). Interactions between the hemiparasitic angiosperm *Rhinanthus minor* and its hosts: from the cell to the ecosystem. Folia Geobot. 40, 217–229 10.1007/BF0280323617008350

[B11] Crawley M. J. (2012). The R Book. Chichester: Wiley 10.1002/9781118448908

[B12] Ewald N. C. John E. A. Hartley S. E. (2011). Responses of insect herbivores to sharing a host plant with a hemiparasite: impacts on preference and performance differ with feeding guild. Ecol. Entomol. 36, 596–604 10.1111/j.1365-2311.2011.01304.x

[B13] Fournier D. A. Skaug H. J. Ancheta J. Ianelli J. Magnussen A. Maunder M. (2012). AD Model builder: using automatic differentiation for statistical inference of highly parameterized complex nonlinear models. Optim. Method Softw. 27, 233–249 10.1080/10556788.2011.597854

[B14] Fox J. Weisberg S. (2011). An {R} Companion to Applied Regression. Thousand Oak, CA: Sage

[B15] Fridley J. D. Grime J. P. Bilton M. (2007). Genetic identity of interspecific neighbours mediates plant responses to competition and environmental variation in a species-rich grassland. J. Ecol. 95, 908–915 10.1111/j.1365-2745.2007.01256.x

[B16] Fritz R. S. (1995). Direct and indirect effects of plant genetic variation on enemy impact. Ecol. Entomol. 20, 18–26 10.1111/j.1365-2311.1995.tb00424.x

[B17] Genung M. A. Schweitzer J. A. Ubeda F. Fitzpatrick B. M. Pregitzer C. C. Felker-Quinn E. (2011). Genetic variation and community change - selection, evolution, and feedbacks. Funct. Ecol. 25, 408–419 10.1111/j.1365-2435.2010.01797.x19414476

[B18] Gibson C. C. Watkinson A. R. (1989). The host range and selectivity of a parasitic plant - *Rhinanthus minor* L. Oecologia 78, 401–406 10.1007/BF00379116 28312588

[B19] Hoagland D. R. Arnon D. I. (1950). The water-culture method for growing plants without soil. Calif. Agr. Exp. Stn. Circ. 347, 1–32

[B20] Irwin R. E. (2006). The consequences of direct versus indirect species interactions to selection on traits: pollination and nectar robbing in *Ipomopsis aggregata*. Am. Nat. 167, 315–328 10.1086/499377 16673341

[B21] Jiang F. Jeschke W. D. Hartung W. (2004). Solute flows from *Hordeum vulgare* to the hemiparasite *Rhinanthus minor* and the influence of infection on host and parasite nutrient relations. Funct. Plant Ecol. 31, 633–643 10.1071/FP03225 32688935

[B22] Johnson M. T. J. (2008). Bottom-up effects of plant genotype on aphids, ants, and predators. Ecology 89, 145–154 10.1890/07-0395.1 18376556

[B23] Johnson M. T. J. Agrawal A. A. (2005). Plant genotype and environment interact to shape a diverse arthropod community on evening primrose (*Oenothera biennis*). Ecology 86, 874–885 10.1890/04-1068

[B24] Kaunisto K. M. Suhonen J. (2013). Parasite burden and the insect immune response: interpopulation comparison. Parasitology 140, 87–94 10.1017/S0031182012001369 22932032

[B25] Klaren C. H. Janssen G. (1978). Physiological changes in the hemiparasite *Rhinanthus serotinus* before and after attachment. Physiol. Plantarum 42, 151–155 10.1111/j.1399-3054.1978.tb01556.x

[B26] Krebs C. J. (2001). Ecology: the Experimental Analysis of Distribution and Abundance. San Francisco, CA: Benjamin Cummings

[B27] Lambrechts L. Halbert J. Durand P. Gouagna L. C. Koella J. C. (2005). Host genotype by parasite genotype interactions underlying the resistance of anopheline mosquitoes to *Plasmodium falciparum*. Malar. J. 4, 3 10.1186/1475-2875-4-3 15644136 PMC548507

[B28] Marvier M. A. (1996). Parasitic plant-host interactions: plant performance and indirect effects on parasite-feeding herbivores. Ecology 77, 1398–1409 10.2307/2265537

[B29] Mutic J. J. Wolf J. B. (2007). Indirect genetic effects from ecological interactions in *Arabidopsis thaliana*. Mol. Ecol. 16, 2371–2381 10.1111/j.1365-294X.2007.03259.x 17561898

[B30] Mutikainen P. Salonen V. Puustinen S. Koskela T. (2000). Local adaptation, resistance, and virulence in a hemiparasitic plant-host plant interaction. Evolution 54, 433–440 10.1111/j.0014-3820.2000.tb00046.x 10937220

[B31] R Core Team (2013). R: A Language and Environment for Statistical Computing. Vienna: R Foundation for Statistical Computing

[B32] Rowntree J. K. Cameron D. D. Preziosi R. F. (2011a). Genetic variation changes the interactions between the parasitic plant-ecosystem engineer *Rhinanthus* and its hosts. Philos. Trans. R Soc. Lond. B Biol. Sci. 366, 1380–1388 10.1098/rstb.2010.0320 21444312 PMC3081574

[B33] Rowntree J. K. Shuker D. M. Preziosi R. F. (2011b). Forward from the crossroads of ecology and evolution. Philos. Trans. R Soc. Lond. B Biol. Sci. 366, 1322–1328 10.1098/rstb.2010.0357 21444306 PMC3081579

[B34] Salvaudon L. Heraudet V. Shykoff J. A. (2005). Parasite-host fitness trade-offs change with parasite identity: genotype-specific interactions in a plant-pathogen system. Evolution 59, 2518–2524 10.1554/05-299.1 16526500

[B35] Schädler M. Brandl R. Kempel A. (2010). Host plant genotype determines bottom-up effects in an aphid-parasitoid-predator system. Entomol. Exp. Appl. 135, 162–169 10.1111/j.1570-7458.2010.00976.x

[B36] Seel W. E. Jeschke W. D. (1999). Simultaneous collection of xylem sap from *Rhinanthus minor* and the hosts *Hordeum* and *Trifolium*: hydraulic properties, xylem sap composition and effects of attachment. New Phytol. 143, 281–298 10.1046/j.1469-8137.1999.00461.x

[B37] Skaug H. Fournier D. Nielsen A. Magnusson A. Bolker B. (2013). Generalized Linear Mixed Models using AD Model Builder. R package version 0.7.7. Available online at: glmmadmb.r-forge.r-project.org

[B38] Spielman D. Brook B. W. Briscoe D. A. Frankham R. (2004). Does inbreeding and loss of genetic diversity decrease disease resistance? Conserv. Genet. 5, 439–448 10.1023/B:COGE.0000041030.76598.cd

[B39] Strauss S. Y. Irwin R. E. (2004). Ecological and evolutionary consequences of multispecies plant-animal interactions. Annu. Rev. Ecol. Evol. Syst. 35, 435–466 10.1146/annurev.ecolsys.35.112202.130215

[B40] Ter Horst C. P. (2010). Evolution in response to direct and indirect ecological effects in pitcher plant inquiline communities. Am. Nat. 176, 675–685 10.1086/657047 20955011

[B41] Tesitel J. Leps J. Vrablova M. Cameron D. D. (2011). The role of heterotrophic carbon acquisition by the hemiparasitic plant *Rhinanthus alectorolophus* in seedling establishment in natural communities: a physiological perspective. New Phytol. 192, 188–199 10.1111/j.1469-8137.2011.03777.x 21627666

[B42] Tétard-Jones C. Kertesz M. A. Gallois P. Preziosi R. F. (2007). Genotype-by-genotype interactions modified by a third species in a plant-insect system. Am. Nat. 170, 492–499 10.1086/520115 17879200

[B43] Thompson J. N. (2013). Relentless Evolution. Chicago, IA: University of Chicago Press 10.7208/chicago/9780226018898.001.0001

[B44] Whitham T. G. Bailey J. K. Schweitzer J. A. Shuster S. M. Bangert R. K. Leroy C. J. (2006). A framework for community and ecosystem genetics: from genes to ecosystems. Nat. Rev. Genet. 7, 510–523 10.1038/nrg1877 16778835

[B45] Whitham T. G. Young W. P. Martinsen G. D. Gehring C. A. Schweitzer J. A. Shuster S. M. (2003). Community and ecosystem genetics: a consequence of the extended phenotype. Ecology 84, 559–573 10.1890/0012-9658(2003)084[0559:CAEGAC]2.0.CO;2

[B46] Whitlock R. Grime J. P. Booth R. Burke T. (2007). The role of genotypic diversity in determining grassland community structure under constant environmental conditions. J. Ecol. 95, 895–907 10.1111/j.1365-2745.2007.01275.x

[B47] Whitlock R. Grime J. P. Burke T. (2010). Genetic variation in plant morphology contributes to the species-level structure of grassland communities. Ecology 91, 1344–1354 10.1890/08-2098.1 20503867

[B48] Wootton J. T. (1994). The nature and consequences of indirect effects in ecological communities. Annu. Rev. Ecol. Syst. 25, 443–466 10.1146/annurev.es.25.110194.002303

[B49] Zytynska S. E. Fay M. F. Penney D. Preziosi R. F. (2011). Genetic variation in a tropical tree species influences the associated epiphytic plant and invertebrate communities in a complex forest ecosystem. Philos. Trans. R Soc. Lond. B Biol. Sci. 366, 1329–1336 10.1098/rstb.2010.0183 21444307 PMC3081567

[B50] Zytynska S. E. Fleming S. Tetard-Jones C. Kertesz M. A. Preziosi R. F. (2010). Community genetic interactions mediate indirect ecological effects between a parasitoid wasp and rhizobacteria. Ecology 91, 1563–1568 10.1890/09-2070.1 20583697

[B51] Zytynska S. E. Frantz L. Hurst B. Johnson A. Preziosi R. F. Rowntree J. K. (2014). Host-plant genotypic diversity and community genetic interactions mediate aphid spatial distribution. Ecol. Evol. 4, 121–131 10.1002/ece3.916 24558568 PMC3925376

