# Supplementary material for: The genetics of indirect ecological effects—plant parasites and aphid herbivores
Source: Front Genet. 2014 Apr 8;5:72. doi: 10.3389/fgene.2014.00072 (PMC3986559; doi:10.3389/fgene.2014.00072)
Supplement: Supplementary file 1 [file DataSheet1.DOCX]

**Table S1a**. Coefficient estimates with standard errors for the fixed effects and variance with standard deviance for the random effects from the generalised linear mixed model with binomial distribution on the number of *Rhinanthus minor* individuals that survived to pre-attachment seedling stage post germination. The model was calculated using glmmADMB in R. The total number of observations was 1230 and the number of random effects (Pots) was 205.

| Coefficients | Estimates | Standard Error |
| --- | --- | --- |
| Intercept | 1.2615 | 0.1703 |
| Diversity (Low) | -0.0072 | 0.2281 |
| Rhinanthus (Somerset) | -0.5070 | 0.2307 |
| Diversity (Low)*Rhinanthus (Somerset) | -0.2335 | 0.3140 |
| Random effect | Variance | Standard deviation |
| Pot | 0.3676 | 0.6063 |

**Table S1b.** Analysis of Deviance Table (Type II tests) for the number of *Rhinanthus minor* individuals that survived to pre-attachment seedling stage post germination. Significant factors (p < 0.05) are noted in **bold**.

| Factor | df | *X^2^* value | p value |
| --- | --- | --- | --- |
| Diversity | 1 | 0.0082 | 0.9280 |
| **Rhinanthus population** | **1** | **4.8655** | **0.0274** |
| Diversity*Rhinanthus | 1 | 0.5529 | 0.4571 |
| Residuals | 1225 |  |  |

**Table S2a.** Coefficient estimates with standard errors for the fixed effects and variance with standard deviance for the random effects from the generalised linear mixed model with binomial distribution on the number of *Rhinanthus minor* individuals that survived post emergence to the post attachment life stage. The model was calculated using glmmADMB in R. The total number of observations was 864 and the number of random effects (Pots) was 202.

| Coefficients | Estimates | Standard Error |
| --- | --- | --- |
| Intercept | 0.6266 | 0.1549 |
| Diversity (Low) | 0.4054 | 0.2179 |
| Rhinanthus (Somerset) | 0.0175 | 0.2278 |
| Diversity (Low)*Rhinanthus (Somerset) | -0.4864 | 0.3193 |
| Random effect | Variance | Standard deviation |
| Pot | 0.1637 | 0.4046 |

**Table S2b.** Analysis of Deviance Table (Type II tests) for the number of *Rhinanthus minor* individuals that survived post emergence to the post attachment life stage. Significant factors (p < 0.05) are noted in **bold**.

| Factor | df | *X^2^* value | p value |
| --- | --- | --- | --- |
| Diversity | 1 | 3.2258 | 0.0725 |
| Rhinanthus population | 1 | 0.0120 | 0.9127 |
| Diversity*Rhinanthus | 1 | 2.3202 | 0.1277 |
| Residuals | 859 |  |  |

**Table S3a.** Coefficient estimates with standard errors for the fixed effects and variance with standard deviance for the random effects from the generalised linear mixed model with binomial distribution on the number of *Rhinanthus minor* individuals that survived post attachment and flowered. The model was calculated using glmmADMB in R. The total number of observations was 500 and the number of random effects (Pots) was 169.

| Coefficients | Estimates | Standard Error |
| --- | --- | --- |
| Intercept | -1.0194 | 2.0813 |
| Diversity (Low) | 0.6206 | 2.1778 |
| Rhinanthus (Somerset) | -1.5599 | 2.2616 |
| Green aphid genotype (H1) | -2.8424 | 2.1884 |
| Brown aphid genotype (HF92a) | -2.2947 | 2.3154 |
| Total number of aphids (covariate) | 0.0150 | 0.0920 |
| Diversity (Low) * Rhinanthus (Somerset) | 3.9915 | 3.2730 |
| Diversity (Low) * Green aphid (H1) | 2.7689 | 3.0059 |
| Rhinanthus (Somerset) * Green aphid (H1) | 3.8654 | 3.4106 |
| Diversity (Low) * Brown aphid (HF92a) | -0.0357 | 3.1354 |
| Rhinanthus (Somerset) * Brown aphid (HF92a) | 6.8858 | 3.5897 |
| Green aphid (H1) * Brown aphid (HF92a) | 4.9732 | 3.1707 |
| Diversity (Low) * Rhinanthus (Somerset) * Green aphid (H1) | -4.9541 | 4.8064 |
| Diversity (Low) * Rhinanthus (Somerset) * Brown aphid (HF92a) | -9.6920 | 5.0917 |
| Diversity (Low) * Green aphid (H1) * Brown aphid (HF92a) | -4.2113 | 4.3680 |
| Rhinanthus (Somerset) * Green aphid (H1) * Brown aphid (HF92a) | -9.6229 | 5.0039 |
| Diversity (Low) * Rhinanthus (Somerset) * Green aphid (H1) * Brown aphid (HF92a) | 14.935 | 7.2913 |
| Random effect | Variance | Standard deviation |
| Pot | 16.5 | 4.062 |

**Table S3b.** Analysis of Deviance Table (Type II tests) for the number of *Rhinanthus minor* individuals that survived post attachment and flowered. Significant factors (p < 0.05) are noted in **bold**.

| Factor | df | *X^2^* value | p value |
| --- | --- | --- | --- |
| Diversity | 1 | 0.0002 | 0.9900 |
| Rhinanthus population | 1 | 1.7147 | 0.1904 |
| Green aphid genotype | 1 | 2.1516 | 0.1424 |
| Brown aphid genotype | 1 | 0.8548 | 0.3552 |
| Total number of aphids (covariate) | 1 | 0.0267 | 0.8702 |
| Diversity * Rhinanthus | 1 | 1.3438 | 0.2464 |
| Diversity * Green aphid | 1 | 0.8352 | 0.3608 |
| Rhinanthus * Green aphid | 1 | 1.2753 | 0.2588 |
| Diversity * Brown aphid | 1 | 0.2116 | 0.4553 |
| Rhinanthus * Brown aphid | 1 | 2.8397 | 0.0920 |
| Green aphid * Brown aphid | 1 | 2.4015 | 0.1212 |
| Diversity * Rhinanthus * Green aphid | 1 | 1.3628 | 0.2430 |
| Diversity * Rhinanthus * Brown aphid | 1 | 3.1003 | 0.0782 |
| Diversity * Green aphid * Brown aphid | 1 | 1.3742 | 0.2411 |
| Rhinanthus * Green aphid * Brown aphid | 1 | 3.6071 | 0.0575 |
| **Diversity * Rhinanthus * Green aphid * Brown aphid** | **1** | **4.1956** | **0.0405** |
| Residuals | 482 |  |  |

**Table S4a.** Coefficient estimates with standard errors for the fixed effects and variance with standard deviance for the random effects from the generalised linear mixed model with negative binomial distribution on the total number of *Rhinanthus minor* buds, flowers and seedpods produced by the plants that flowered. The model was calculated using glmmADMB in R. The total number of observations was 216 and the number of random effects (Pots) was 95.

| Coefficients | Estimates | Standard Error |
| --- | --- | --- |
| Intercept | 1.9960 | 0.3034 |
| Diversity (Low) | 0.4278 | 0.3101 |
| Rhinanthus (Somerset) | 0.3420 | 0.3400 |
| Green aphid genotype (H1) | 0.4052 | 0.3692 |
| Brown aphid genotype (HF92a) | -0.0822 | 0.3796 |
| Total number of aphids (covariate) | 0.0154 | 0.0135 |
| Diversity (Low) * Rhinanthus (Somerset) | -0.2495 | 0.4412 |
| Diversity (Low) * Green aphid (H1) | -0.3247 | 0.4676 |
| Rhinanthus (Somerset) * Green aphid (H1) | -0.4427 | 0.5466 |
| Diversity (Low) * Brown aphid (HF92a) | -0.3099 | 0.5015 |
| Rhinanthus (Somerset) * Brown aphid (HF92a) | 0.1988 | 0.4915 |
| Green aphid (H1) * Brown aphid (HF92a) | 0.1718 | 0.5229 |
| Diversity (Low) * Rhinanthus (Somerset) * Green aphid (H1) | 0.3909 | 0.7035 |
| Diversity (Low) * Rhinanthus (Somerset) * Brown aphid (HF92a) | 0.3006 | 0.6881 |
| Diversity (Low) * Green aphid (H1) * Brown aphid (HF92a) | 0.0854 | 0.6758 |
| Rhinanthus (Somerset) * Green aphid (H1) * Brown aphid (HF92a) | -0.8003 | 0.7396 |
| Diversity (Low) * Rhinanthus (Somerset) * Green aphid (H1) * Brown aphid (HF92a) | 0.1342 | 0.9752 |
| Random effect | Variance | Standard deviation |
| Pot | 6.9 x 10^-8^ | 0.0003 |

**Table S4b.** Analysis of Deviance Table (Type II tests) for the total number of *Rhinanthus minor* buds, flowers and seedpods produced by the plants that flowered.

| Factor | df | *X^2^* value | p value |
| --- | --- | --- | --- |
| Diversity | 1 | 1.8749 | 0.1709 |
| Rhinanthus population | 1 | 0.9769 | 0.3230 |
| Green aphid genotype | 1 | 1.1770 | 0.2780 |
| Brown aphid genotype | 1 | 0.0379 | 0.8456 |
| Total number of aphids (covariate) | 1 | 1.2976 | 0.2546 |
| Diversity * Rhinanthus | 1 | 0.3269 | 0.5675 |
| Diversity * Green aphid | 1 | 0.4827 | 0.4872 |
| Rhinanthus * Green aphid | 1 | 0.6114 | 0.4343 |
| Diversity * Brown aphid | 1 | 0.3748 | 0.5404 |
| Rhinanthus * Brown aphid | 1 | 0.1487 | 0.6998 |
| Green aphid * Brown aphid | 1 | 0.1005 | 0.7513 |
| Diversity * Rhinanthus * Green aphid | 1 | 0.3048 | 0.5809 |
| Diversity * Rhinanthus * Brown aphid | 1 | 0.1913 | 0.6618 |
| Diversity * Green aphid * Brown aphid | 1 | 0.0159 | 0.8995 |
| Rhinanthus * Green aphid * Brown aphid | 1 | 1.1655 | 0.2803 |
| Diversity * Rhinanthus * Green aphid * Brown aphid | 1 | 0.0189 | 0.8905 |
| Residuals | 197 |  |  |

**Table S5a.** Coefficient estimates with standard errors for the fixed effects and variance with standard deviance for the random effects from the general linear mixed model on Barley shoot dry weight. The model was calculated using lme4 in R. The total number of observations was 1654 and the number of random effects (Pots) was 277.

| Coefficients | Estimates | Standard Error |
| --- | --- | --- |
| Intercept | 0.8135 | 0.0317 |
| Diversity (Low) | 0.0039 | 0.0422 |
| Rhinanthus (Presence) | -0.1093 | 0.0393 |
| Green aphid genotype (H1) | -0.0024 | 0.0438 |
| Brown aphid genotype (HF92a) | -0.0204 | 0.0438 |
| Total number of aphids (covariate) | 0.0374 | 0.0028 |
| Number of Rhinanthus plants (covariate) | -0.0413 | 0.0064 |
| Diversity (Low) * Rhinanthus (Presence) | 0.0249 | 0.0529 |
| Diversity (Low) * Green aphid (H1) | -0.0365 | 0.0599 |
| Rhinanthus (Presence) * Green aphid (H1) | 0.0207 | 0.0553 |
| Diversity (Low) * Brown aphid (HF92a) | 0.0043 | 0.0602 |
| Rhinanthus (Presence) * Brown aphid (HF92a) | 0.0396 | 0.0547 |
| Green aphid (H1) * Brown aphid (HF92a) | 0.0178 | 0.0626 |
| Diversity (Low) * Rhinanthus (Presence) * Green aphid (H1) | 0.0137 | 0.0757 |
| Diversity (Low) * Rhinanthus (Presence) * Brown aphid (HF92a) | 0.0458 | 0.0758 |
| Diversity (Low) * Green aphid (H1) * Brown aphid (HF92a) | 0.0577 | 0.0854 |
| Rhinanthus (Presence) * Green aphid (H1) * Brown aphid (HF92a) | -00139 | 0.0788 |
| Diversity (Low) * Rhinanthus (Presence) * Green aphid (H1) * Brown aphid (HF92a) | -0.0903 | 0.1076 |
| Random effect | Variance | Standard deviation |
| Pot | 0.0061 | 0.0779 |
| Residual | 0.0326 | 0.1805 |

**Table S5b.** Analysis of Deviance Table (Type II tests) for Barley shoot dry weight. Significant factors (p < 0.05) are noted in **bold**.

| Factor | df | *X^2^* value | p value |
| --- | --- | --- | --- |
| Diversity | 1 | 2.9781 | 0.0844 |
| **Rhinanthus presence** | **1** | **17.2517** | **3.3x10^-5^** |
| Green aphid genotype | 1 | 0.1268 | 0.7218 |
| **Brown aphid genotype** | **1** | **4.1549** | **0.04151** |
| **Total number of aphids (covariate)** | **1** | **184.6013** | **<2.2x10^-16^** |
| **Number of Rhinanthus plants (covariate)** | **1** | **42.1827** | **8.3x10^-11^** |
| Diversity * Rhinanthus | 1 | 1.3974 | 0.2372 |
| Diversity * Green aphid | 1 | 1.1508 | 0.2834 |
| Rhinanthus * Green aphid | 1 | 0.0175 | 0.8948 |
| Diversity * Brown aphid | 1 | 1.6684 | 0.1965 |
| Rhinanthus * Brown aphid | 1 | 1.4597 | 0.2270 |
| Green aphid * Brown aphid | 1 | 0.1379 | 0.7104 |
| Diversity * Rhinanthus * Green aphid | 1 | 0.3346 | 0.5630 |
| Diversity * Rhinanthus * Brown aphid | 1 | 0.0004 | 0.9835 |
| Diversity * Green aphid * Brown aphid | 1 | 0.0002 | 0.9881 |
| Rhinanthus * Green aphid * Brown aphid | 1 | 1.3396 | 0.2471 |
| Diversity * Rhinanthus * Green aphid * Brown aphid | 1 | 0.7036 | 0.4016 |

**Table S6.** Analysis of Deviance Table (Type II tests) for Barley shoot dry weight from the low diversity pots. The total number of observations was 874 and the number of random effects (Pots) was 147. Variance and standard errors for the random factor are shown at the bottom of the table. Significant factors (p < 0.05) are noted in **bold**.

| Factor | df | *X^2^* value | p value |
| --- | --- | --- | --- |
| **Barley genotype** | **5** | **74.0674** | **1.5x10^-14^** |
| **Rhinanthus presence** | **1** | **5.2347** | **0.0221** |
| Green aphid genotype | 1 | 0.3962 | 0.5291 |
| **Brown aphid genotype** | **1** | **5.7946** | **0.0161** |
| **Total number of aphids (covariate)** | **1** | **155.5320** | **2.2x10^-16^** |
| **Number of Rhinanthus plants (covariate)** | **1** | **33.4314** | **7.4x10^-9^** |
| Barley * Rhinanthus | 5 | 3.0274 | 0.6968 |
| Barley * Green aphid | 5 | 1.9823 | 0.8516 |
| Rhinanthus * Green aphid | 1 | 0.1691 | 0.6809 |
| Barley * Brown aphid | 5 | 4.8442 | 0.4352 |
| Rhinanthus * Brown aphid | 1 | 0.9966 | 0.3182 |
| Green aphid * Brown aphid | 1 | 0.2145 | 0.6432 |
| Barley * Rhinanthus * Green aphid | 5 | 7.3528 | 0.1957 |
| Barley * Rhinanthus * Brown aphid | 5 | 2.4064 | 0.7905 |
| **Barley * Green aphid * Brown aphid** | **5** | **12.4893** | **0.0287** |
| Rhinanthus * Green aphid * Brown aphid | 1 | 2.6509 | 0.1035 |
| Barley * Rhinanthus * Green aphid * Brown aphid | 5 | 10.6250 | 0.0593 |
| Random effect | Variance | | Standard deviation |
| Pots | 0.0065 | | 0.0803 |
| Residual | 0.0158 | | 0.1257 |

**Table S7.** Analysis of Deviance Table (Type II tests) for Barley shoot dry weight from the high diversity pots. The total number of observations was 780 and the number of random effects (Pots) was 130. Variance and standard errors for the random factor are shown at the bottom of the table. Significant factors (p < 0.05) are noted in **bold**.

| Factor | df | *X^2^* value | p value |
| --- | --- | --- | --- |
| **Barley genotype** | **5** | **504.5081** | **<2.2x10^-16^** |
| **Rhinanthus presence** | **1** | **21.5430** | **3.5x10^-6^** |
| Green aphid genotype | 1 | 1.2211 | 0.2691 |
| Brown aphid genotype | 1 | 0.2774 | 0.5984 |
| **Total number of aphids (covariate)** | **1** | **90.6657** | **<2.2x10^-16^** |
| **Number of Rhinanthus plants** | **1** | **17.7284** | **2.5x10^-5^** |
| Barley * Rhinanthus | 5 | 2.8433 | 0.7241 |
| Barley * Green aphid | 5 | 5.3631 | 0.3732 |
| Rhinanthus * Green aphid | 1 | 0.3390 | 0.5604 |
| **Barley * Brown aphid** | **5** | **22.7708** | **0.0004** |
| Rhinanthus * Brown aphid | 1 | 0.9186 | 0.3379 |
| Green aphid * Brown aphid | 1 | 0.0045 | 0.9464 |
| Barley * Rhinanthus * Green aphid | 5 | 2.4059 | 0.7906 |
| Barley * Rhinanthus * Brown aphid | 5 | 3.6926 | 0.5945 |
| Barley * Green aphid * Brown aphid | 5 | 5.6008 | 0.3470 |
| Rhinanthus * Green aphid * Brown aphid | 1 | 0.1059 | 0.7448 |
| Barley * Rhinanthus * Green aphid * Brown aphid | 5 | 7.5086 | 0.1855 |
| Random effect | Variance | | Standard deviation |
| Pots | 0.0038 | | 0.0620 |
| Residual | 0.0286 | | 0.1692 |
